# Assessing Postoperative Pain in Patients Who Underwent Total Knee Arthroplasty Using an Automated Self-Logging Patient-Reported Outcome Measure Collection Device: Retrospective Cohort Study

**DOI:** 10.2196/65271

**Published:** 2025-07-10

**Authors:** Prabjit Ajrawat, Blaine Price, Daniel Gooch, Rudolf Serban, Ruqaiya Al-Habsi, Oliver Pearce

**Affiliations:** 1 University of Buckingham Medical School, Buckingham, United Kingdom; 2 School of Computing and Communications, The Open University, Jenny Lee Building, Walton Hall, Milton Keynes, MK7 6AA, United Kingdom, +441908858234; 3 Trauma and Orthopaedics Department, Milton Keynes University Hospital, Milton Keynes, United Kingdom; 4 Trauma and Orthopaedics Department, Barts Health NHS Trust, London, United Kingdom

**Keywords:** electronic patient-reported outcome measure, total knee arthroplasty, tourniquet, PainPad, knee arthroplasty, automated self-logging, collection device, retrospective cohort study, patient-reported outcome, quality of care, assessment, data collection, postoperative, feasibility, effectiveness, electronic patient report, patient-centered care, innovative technology

## Abstract

**Background:**

Patient-reported outcome measures (PROMs) are tools for assessing symptoms and the quality of care. Despite their growing use, conventional data collection methods limit widespread PROM implementation. In orthopedics, pain is a frequent patient complaint and a common PROM, especially following total knee arthroplasty (TKA). Although TKA is generally successful, some patients still report postoperative pain, potentially due to tourniquet use. Using an improved PROM data-gathering technique may help to address tourniquet use during a TKA procedure and its impact on postoperative pain. The PainPad, an automated self-logging device, was developed to capture patient pain levels accurately.

**Objective:**

The aim of the study is to assess the feasibility and effectiveness of the PainPad device in quantifying in-hospital postoperative pain following TKA with or without tourniquet use.

**Methods:**

A retrospective study with 234 patients who underwent TKA from 2018 to 2021 at Milton Keynes University Hospital was conducted. Patients were categorized as receiving TKA with an intraoperative tourniquet (tourniquet group) or TKA without a tourniquet (nontourniquet group). Postoperative pain during the first 24 hours was self-reported every 2 hours using the PainPad device. From both groups, data on hospital length of stay, total tourniquet time, and the presence of postoperative deep vein thrombosis were also collected.

**Results:**

There were 115 TKAs with tourniquets (72/115, 62.6% female patients; mean age 69.26, SD 9.93 years) and 119 TKAs without tourniquets (91/119, 76.4% female patients; mean age 70.97, SD 9.01 years). When assessing 24-hour mean postoperative pain scores, the PainPad device data indicated no significant difference (*P*=.53; 95% CI −0.76 to 0.39) between the tourniquet (mean pain score 3.31, SD 2.34) and nontourniquet groups (mean pain score 3.12, SD 2.15). There was no correlation between tourniquet times and the pain scores retrieved from the PainPad device. A subgroup analysis comparing longer (>90 minutes) versus shorter (<90 minutes) tourniquet times showed no significant difference in terms of pain and length of stay.

**Conclusions:**

The PainPad device is a feasible and effective method for collecting and evaluating in-hospital postoperative pain following TKA, allowing for the quantification of individual pain levels. This study aligns with the current health care trend toward leveraging innovative technologies and personalized data to enhance patient-centered care.

## Introduction

Patient-reported outcome measures (PROMs) are tools that have become increasingly used to systematically assess symptom progression, satisfaction, and the overall quality of care provided from the patient’s perspective [1,2]. Originally designed for research purposes, incorporating PROMs into clinical care represents a transition toward a more evidence-based and patient-centered health care system [3]. Implementing PROMs enables patients to quantify their subjective experience and allows physicians to monitor symptoms and make informed treatment decisions when combined with other objective measures.

Among surgical specialties, PROMs have been most extensively administered in orthopedics [4]. Since April 2009, the National Health Service (NHS) mandated all publicly funded health care providers of inpatient departments to collect PROMs for certain elective procedures, including hip and knee arthroplasties [5]. Despite the increasing use and evidence supporting PROMs, widespread implementation still faces several challenges among orthopedic practices. The additional time, cost, and administrative burden along with the risks of low patient participation, data errors, and incorrect interpretation have been frequently cited as limitations in integrating PROMs into surgical practices [3,5,6]. These limitations may be attributed to the current inefficiencies of conventional paper-based data collection methods. As initiatives strive to sustain PROM use, innovative data collection technologies tailored to the complexities of surgical workflows will be needed to alleviate these logistic and administrative constraints.

Within orthopedic practices, in-hospital postoperative pain is a regularly assessed PROM following surgical interventions, as it is a frequently cited patient complaint [7,8]. While some PROMs can be objectively measured, pain is inherently subjective and difficult to accurately capture. Current methods, such as patient pain diaries, are often prone to inaccuracies due to illegible handwriting, incomplete data, and recall bias [9-11]. Additionally, distributing, collecting, and processing paper-based PROM methods require significant administrative efforts and costs [12]. The manual data handling and entry further increase the risk of transcription errors, compromising the reliability of the collected data. The physical forms of paper-based methods also require substantial storage space, which can limit the timely retrieval of patient data [12]. The emergence of electronic devices and platforms presents an alternative approach to collecting pain-related data. Evidence suggests that electronic data collection methods may be more accurate, provide greater compliance, and yield fewer errors compared to conventional methods [13,14].

Even with total knee arthroplasty (TKA) being one of the most successful orthopedic procedures, 20% of patients were unsatisfied due to their postoperative pain levels [15]. A potential cause for this pain may be due to tourniquet use during a TKA procedure; however, this topic remains controversial among orthopedic surgeons. However, by using an improved PROM data-gathering technique, it may help to address the conflicting topic of tourniquet use during a TKA procedure and its impact on postoperative pain.

The PainPad is a handheld automated self-logging device that was developed to capture patient pain levels [11]. While other electronic pain collection methods have focused on remote monitoring in outpatient settings, the PainPad device was designed for inpatient hospital use to provide instantaneous postsurgical pain evaluation [11]. Thus, to showcase the clinical utility and effectiveness of assessing postoperative pain, the purpose of this study was to use the PainPad device to determine if tourniquet use during TKA has a detrimental effect on postoperative pain levels in the context of a modern multimodal pain pathway and early mobilization.

## Methods

### Ethical Considerations

This research was approved under Integrated Research Application System (229503) and the Open University Human Research Ethics Committee (17/LO/1404). Informed consent was provided by participants, and data were deidentified.

### Study Design and Patients

This retrospective cohort study was conducted at Milton Keynes University Hospital (MKUH), United Kingdom, and reviewed 234 patients who underwent TKA. Patients undergoing a primary unilateral TKA between 2018 and 2021 at MKUH were included in the study. Any patients undergoing revision TKA were excluded.

### Surgical Procedure and Postoperative Care

All cases were performed by 7 arthroplasty consultants at MKUH, and all patients underwent the multimodal analgesia program protocol for their inpatient rehabilitation. This modern multimodal analgesia protocol was specifically designed to enhance the recovery process, decrease the amount and frequency of opioid use, and reduce the length of stay (LOS) [16]. All patients were assessed preoperatively by the physiotherapy team to collect PROMs and range of movement.

Patients were categorized as receiving TKA with an intraoperative tourniquet (tourniquet group) or TKA without a tourniquet (nontourniquet group). For patients receiving the tourniquet, a conical tourniquet (85 cm long×8.5 cm wide) was inflated to 300 mm Hg and kept inflated from the beginning of the operation to the end of the procedure. This duration was considered the tourniquet time. Aside from tourniquet use, all TKAs were performed through the same standard surgical approach, and all patients received the same postoperative rehabilitation protocol.

### The PainPad Device

**Figure 1.**
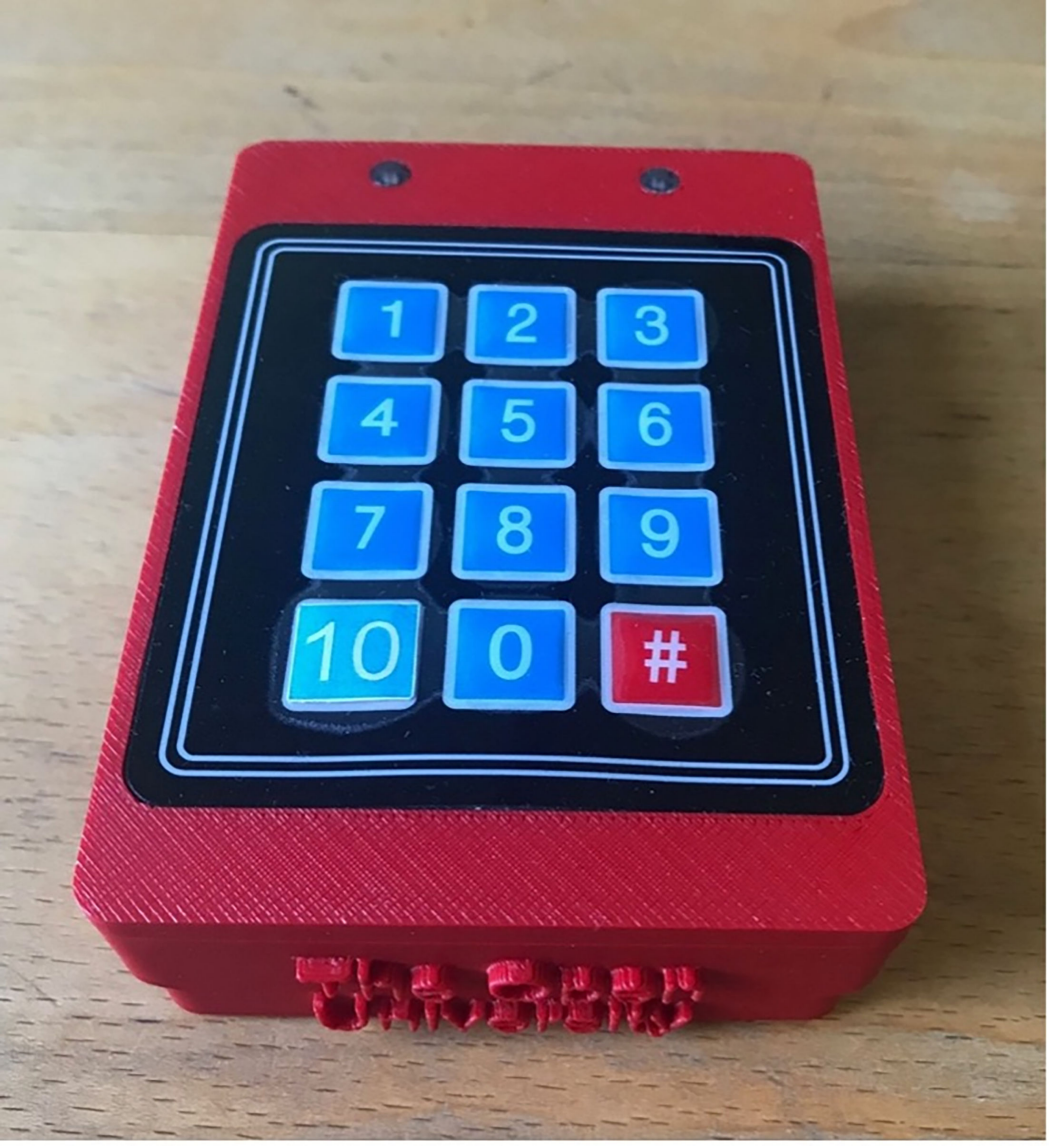
The PainPad device (9.5×6.5×3 cm) is a 3D-printed rectilinear box made of acrylonitrile butadiene styrene (ABS) plastic. It features a red and green surface-mounted LED light on the internal circuit board, which projects through light tubes on the surface, and a 3×4 membrane keypad recessed into the front face. Beneath the ABS shell is a 70-dB speaker that emits sounds prompting the user to self-report their pain level by pressing a button (0‐10), which beeps when pressed. The device also plays distinct sounds to signal success or failure when clinicians assign it to a patient.

Patients reported their postoperative pain levels using an automated self-logging device named “PainPad” (Figure 1). A previous study has outlined PainPad’s design process and user testing with patients and hospital staff [11]. The handheld PainPad device (9.5×6.5×3 cm) consists of a large keypad allowing patients to rate their pain on a scale of 0‐10 (0=no pain and 10=worst pain imaginable), mimicking the visual analog scale (VAS). Patients were shown how to enter their pain level using the PainPad device. The physical box-like design of the PainPad allowed for easy and quick disinfectant and sanitary practices by hospital personnel. The PainPad device consists of 2 LEDs and a beeper to notify patients to enter their pain score every 2 hours to ensure compliance with pain logging [11]. These pain scores were then linked to the patient via their unique identifier. The combination of PainPad’s pain database and the nurse’s entered pain scores on the electronic patient record was combined to gain a maximal number of pain scores for each inpatient episode for analysis.

### Outcome Measures

The primary outcome of this study was the self-reported 24-hour postoperative pain scores (mean, maximum, and minimum) on the PainPad device in both tourniquet and nontourniquet groups. Secondary outcomes included measuring hospital LOS, total tourniquet time, and the presence of postoperative deep vein thrombosis (DVT) between both tourniquet and nontourniquet groups.

### Statistical Analysis

Descriptive statistics were used to analyze patient demographics and general trends in outcome measures. Two-tailed *t* tests were used to determine the differences in patient demographics, 24-hour postoperative pain scores from the PainPad device, and hospital LOS between tourniquet and nontourniquet patients. Additionally, 2-tailed *t* tests were used to assess PainPad pain data and hospital LOS between a subgroup of patients with longer (>90 minutes) or shorter (<90 minutes) tourniquet times. The Spearman rank correlation was used to compare the tourniquet time with PainPad pain scores and hospital LOS. For our statistical analysis, the 2-tailed *t* test was suitable in comparing 2 groups of patients (ie, with and without tourniquet), with a Spearman rank determining a correlation between tourniquet use and pain scores. Our data met the criteria for both the 2-tailed *t* test (independence of observations, approaching normal distribution, and homogeneity of variances assessed through the Levene test) and Spearman rank (monotonic, paired, and interval data). All statistical testing was conducted using the SPSS software (version 21; IBM Corp).

## Results

A total of 234 consecutive patients who underwent TKA (n=163, 70% female patients) were included (mean age 69.26, SD 9.93 years) and 119 underwent TKA without a tourniquet (91/119, 76.4% female patients; mean age 70.97, SD 9.01 years). Regarding patient demographics, there was a significant difference in female sex (*P*=.02) favoring the nontourniquet group (Table 1). The median hospital LOS was 2 (IQR 1‐18) days, and there was no significant difference in hospital LOS between the 2 groups (*P*=.22; 95% CI −0.24 to 1.04). From the cohort, only 1 of 234 patients experienced a DVT event, which was from the nontourniquet group.

**Table 1.** Characteristics of included patients who underwent total knee arthroplasty using the PainPad device in the nontourniquet group and tourniquet groups.

Patient characteristics	Nontourniquet (n=119)	Tourniquet (n=115)	*P* value
Sex, n (%)			.02
Male	28 (23.5)	43 (37.3)	
Female	91 (76.4)	72 (62.6)
Age (years), mean (SD)	70.97 (9.01)	69.26 (9.93)	.17

The PainPad device was used to assess patient-reported postoperative pain between the 2 groups. When assessing 24-hour mean postoperative pain scores, the PainPad device data indicated that there was no significant difference between the tourniquet (mean pain score 3.31, SD 2.34) and nontourniquet group (mean pain score 3.12, SD 2.15; *P*=.53; 95% CI −0.76 to 0.39). The PainPad device data further suggested no significant difference in the maximum pain score 24 hours postoperatively between the tourniquet (mean maximum pain score 6.34, SD 2.81) and nontourniquet group (mean maximum pain score 6.12, SD 2.87; *P*=.55; 95% CI −0.95 to 0.51). There was also no significant difference in the minimum pain score 24 hours postoperatively between the tourniquet (mean minimum pain score 0.66, SD 1.72) and nontourniquet (mean minimum pain score 1.03, SD 2.22; *P*=.15; 95% CI −0.89 to 0.13).

Tourniquet time was recorded from 110 of 115 patients with a median time of 73 (IQR 19‐120) minutes. There was no correlation between tourniquet time and hospital LOS (Spearman ρ=−0.09; *P*=.38). When correlating tourniquet time with 24-hour postoperative pain scores retrieved from the PainPad device, there were no correlations for the mean (Spearman ρ=−0.01; *P*=.90), maximum (Spearman ρ=−0.18; *P*=.07), and minimum pain scores (Spearman ρ=0.12; *P*=.20). In correlating the VAS with 24-hour postoperative mean pain scores, there was a very low correlation (*r*=0.139) and an extremely low correlation with maximum pain score 24 hours postoperatively (*r*=0.078). This indicates that preoperative and postoperative pain were not correlated. A subgroup analysis of patients with longer tourniquet times (>90 minutes) and shorter tourniquet times (<90 minutes) was also evaluated. From this, there were no significant differences between longer and shorter tourniquet times in terms of hospital LOS and self-reported pain data as collected from the PainPad device (Table 2).

**Table 2.** Comparison of recorded PainPad postoperative pain levels and hospital length of stay (LOS) between long duration (>90 minutes) and short duration (<90 minutes) tourniquet groups.

Outcomes	Long duration tourniquet(n=91), mean (SD)	Short duration tourniquet(n=19), mean (SD)	*P* value	95% CI
Minimum pain	0.86 (2.01)	2.16 (3.04)	.09	−2.81 to 0.21
Maximum pain	6.29 (2.87)	6.37 (2.79)	.91	−1.54 to 1.37
Mean pain	3.14 (2.23)	4.00 (2.86)	.23	−2.30 to 0.58
Hospital LOS (days)	2.74 (2.49)	2.53 (1.81)	.67	−0.49 to 1.21

## Discussion

### Principal Findings

PROMs represent a standardized method for monitoring patient symptoms. The value of monitoring patients’ self-reported pain levels during the first hours in the postoperative period is essential to ensure optimal analgesia and patient satisfaction [17,18]. This study provides evidence that the PainPad, an automated self-logging PROM device, is feasible in collecting pain levels after a major orthopedic surgical intervention. Since our institution uses a modern multimodal analgesia protocol to enhance the recovery process and limit the use of postoperative opioids, the PainPad device provided surgeons with real-time pain analytics within the 24-hour postoperative period to ensure that appropriate analgesia and prompt intervention were administered.

PROMs have become increasingly important in the context of orthopedics, as many surgical interventions aim to improve subjective patient outcomes such as pain. Incorporating PROMs, such as pain logging, into routine care can harness critical patient information to improve clinical decision-making and overall support patient-centered care. Generally, self-reported pain logging in an outpatient clinic setting is completed by paper-based methods in the form of questionnaires, forms, or patient diaries [10]. In contrast, during an inpatient hospital stay, nursing staff typically collect pain levels at regular intervals through monitoring or by having patients complete a numerical pain rating scale [19-21]. However, these methods are often time-consuming and impose additional burdens on clinical staff [11]. In some instances, staffing limitations can sometimes prevent sufficiently frequent pain log entries [11]. Additionally, studies have indicated that pain levels recorded by nursing staff are often incomplete and potentially inaccurate, as patients may feel reluctant to fully express their pain [20,22]. Orthopedic surgeons have also expressed various logistical and technical challenges in collecting pain levels and other PROMs. For instance, surgeons indicated that the additional administrative tasks involved in data collection and the prevalence of missing data along with poor visual display and the complexity of completing PROMs hindered their ability to administer PROMs to their patients [6]. Collectively, these limitations of conventional paper-based methods coupled with the earlier-mentioned barriers further emphasize the need for innovative technologies to seamlessly integrate self-logging of pain and PROMs.

Currently, the NHS PROM program administers paper-based collection methods, incurring an annual cost of US $1.1 million [23]. However, as the NHS transitions toward a paperless system and the PROM program expands, innovative technologies will be required to reduce costs and streamline data administration [24]. Studies have shown that electronic patient-reported outcome measure (ePROM) tools and devices are equivalent to their original paper-based counterpart [25,26]. With regard to paper-based methods, poor response rate is a limiting factor in assessing PROMs [27,28]. Yet, administrating ePROM tools has been shown to circumvent poor response rates, as automation permits outcomes to be collected at the scheduled intervals. Gurland et al [29] showed that administering ePROMs on a tablet to 103 patients who are undergoing surgery resulted in a 96% response rate in comparison to 25% with a paper-based collection method. Furthermore, ePROMs have the potential to enhance patient-physician communication by offering real-time pain tracking and avoiding possible recall bias [30]. The practicality of ePROM modalities, along with their advantages of automated data capture, lower long-term costs, faster completion times, and reduced administrative errors [12], demonstrates the potential role of ePROM tools within the NHS to ensure widespread data capturing.

Most self-logging pain and ePROM technologies are designed for remote or at-home monitoring. In contrast, the PainPad was developed as an automated device to enable patients to provide their pain ratings during their hospital stay. Previous research with the PainPad showed that it improved the frequency and compliance of self-reported pain logging among inpatients recovering from ambulatory orthopedic surgery compared to pain scores reported by nursing staff [11]. The PainPad offers a convenient user experience for both patients and staff. It was designed with patient feedback, ensuring an easy-to-use interface. In one study, authors noted that 29% of patients had difficulties completing the paper-based VAS form due to visual impairment and physical restriction [31].

The PainPad device has several advantages over other pain assessment methods in a hospital setting. Paper-based or verbal pain assessments can be limited by patient fatigue, cognitive impairment, and verbal communication difficulties and often require manual transcription that could lead to errors [32,33]. Mobile apps and tablets often require patients to navigate various user interfaces and provide typed or touched responses on a touchscreen that may be difficult for older patients, those with dexterity issues, or those experiencing severe postsurgical pain [34]. Other mobile apps such as the PainChek app use facial expressions for pain assessment; however, these tend to be used in long-term care settings for patients with moderate to severe dementia. Unlike other ePROM methods, the lightweight PainPad features larger buttons that make it accessible for those with mobility or vision issues, and the visual cues it provides minimize response errors and ensure accurate data capture. Additionally, the physical device allows patients who are unable to verbalize their pain to report it accurately. Incorporating tactile, auditory, and visual senses into the device is believed to minimize typical patient errors associated with paper-based or single-dimensional methods, thereby providing a more standardized assessment of individualized pain levels. This user-friendly interface minimizes the risk of noncompliance while ensuring immediate, real-time accurate pain logging, making it more suitable for hospital settings where rapid pain assessment is critical. Given the importance of hospital infection control, the device was designed for easy disinfection within the hospital environment and to seamlessly integrate into clinical workflows [11]. However, a drawback of the PainPad is that it has not been assessed in postsurgical pediatric patients, where adaptations may be required.

In our study, most were female participants, and research has shown that pain perception differs between sexes. Female participants often experience higher pain sensitivity and report higher postoperative pain scores compared to male participants following TKA [35]. Additionally, patient age may also influence pain perception, as aging reduces pain sensitivity for lower pain intensities [36]. These sex and age-based differences in pain perception may influence how patients interact with and report pain using the PainPad device. Given these differences in pain perception, it is ideal for patients to self-report their pain at its most intense moments using accessible and patient-friendly tools. Prior research suggests that older patients might prefer alternative methods for self-logging their PROMs or struggle with handheld technologies [12,37,38]. Research has shown that younger patients have a greater preference for ePROM methods [12,38-40]. However, the mean age in our study was 69.2 years, indicating that older patients can effectively use this device. When comparing the PainPad interface to 2 tablet-based alternatives, older adults preferred the tangible PainPad interface for reporting their pain in the hospital setting [11].

In this study, we compared tourniquet use in TKA to showcase the clinical utility and feasibility of the PainPad device in collecting pain levels. Using tourniquets in TKA remains a topic of considerable debate. Some orthopedic surgeons advocate for their use, citing advantages such as improved surgical field visualization, enhanced cementation through increased interdigitation, reduced intraoperative blood loss, and shorter operative times [41]. On the other hand, studies have indicated a slightly elevated risk of DVT, quadriceps weakness, nerve injury, decreased range of movement, higher transfusion rate, longer LOS, and increased postoperative pain [42-49].

Our results suggest that the use of a tourniquet during TKA procedures has no impact on the postoperative VAS score in the context of a modern multimodal pain pathway with early mobilization. Furthermore, there was no significant difference between patients with a tourniquet for <90 minutes and those with a tourniquet for >90 minutes with regard to postoperative pain and hospital LOS. These findings contrast previous reports that demonstrated increased pain among patients who underwent TKA with a tourniquet [50,51]. However, this can be attributed to the predate use of modern multimodal analgesia protocols and early mobilization pathways, which aim to reduce postoperative pain levels and hospital LOS, increase patient mobility, and limit opioid analgesia use [52]. Moreover, the absence of precise PROM data collection methods may have impeded and further exacerbated the true efficacy of this technique. By providing patients with the self-logging PainPad device, we were able to gather sufficient data to demonstrate that tourniquet use is associated with positive patient outcomes, including effective pain management. By making a digital self-logging of PROMs more accessible, we suggest that better evidence will be available to improve other surgical and clinical procedures.

### Limitations

There are limitations to this study that need to be considered. First, this was a retrospective study in a single academic center with 234 patients who underwent TKA, which limits the generalizability of our findings. The retrospective nature of our study may have also potentially introduced inherent selection, recall, and ascertainment biases, as the patient inclusion was based on available data from our institution’s database. However, since the PainPad device collects pain scores in an instantaneous manner, this would ideally limit recall bias. Furthermore, our statistical analysis indicated no significant differences in major baseline confounding variables between groups.

Second, patient comorbidities and preoperative pain data were not collected, which may influence postoperative pain levels. Third, TKA procedures were completed by 7 orthopedic consultants, which may contribute to discrepancies in operative technique. However, all surgeons followed the same postoperative pain management protocol. Finally, this study only assessed 24-hour postoperative pain; however, within the 24 hours, there were multiple self-reported pain entries from patients (ie, completed bihourly) using the PainPad device. Further, large-scale studies that include various acute and chronic patient populations with diverse demographics (ie, pediatric patients) along with appropriate control groups will help ensure the generalizability of the results. Additionally, it will be important to directly evaluate the cost-effectiveness of using the PainPad device in a clinical setting.

### Conclusions

The PainPad device is a feasible and effective method for collecting and evaluating in-hospital postoperative pain following TKA, allowing for precise quantification of individual pain levels. This study aligns with the current health care trend toward leveraging innovative technologies and personalized data to enhance patient-centered care.

## Abbreviations

DVT: deep vein thrombosis
ePROM: electronic patient-reported outcome measure
LOS: length of stay
MKUH: Milton Keynes University Hospital
NHS: National Health Service
PROM: patient-reported outcome measure
TKA: total knee arthroplasty
VAS: visual analog scale
